# Rheumatism and Gout

**Published:** 1906-01-06

**Authors:** 


### PROGRESS IN MEDICINE AND SURGERY.

### RHEUMATISM AND GOUT.

(*Continued from page* 219.)

**A Diet Free from Purins.**—Whether such a diet is useful by promoting intestinal asepsis, or whether it decreases the uric acid in the blood, it is often of use in gouty, rheumatic and neurotic conditions. W. A. Potts reports certain cases of very chronic character which were restored to health by its use. As is well known, such a diet consists of milk, cheese, and butter, bread, cereal foods, and fruits, while meat, fish, tea, coffee, mushrooms, asparagus, and the pulses are excluded. It seems that such a diet, when commenced and carried out carefully, is really capable of sustaining not only a sedentary, but a severely laborious life. The change should be made slowly and gradually, not more than one meal at a time being altered, and any constipation or indigestion resulting at first being carefully treated. Its advocates point out that it need not be purely vegetarian, and claim that headaches, colds, rheumatism, and constipation are got rid of permanently. Whether desirable for the ordinary man or not, and whether it could or could not eliminate gout, they have at any rate shown that it is a possible mode of life, and therefore applicable to certain classes of patients who do not react to ordinary methods. Chalmers Watson[12] points out that an extraordinary revolution in the opposite direction has been taking place in this country during the last 50 years, at least so far as the imports show, imported meat having increased from 3 lbs. per head to 50 lbs. However, the increase of tea, which was equally rapid in the earlier part of the period, has been very slight for the last 20 years, and coffee has of late actually decreased; while sugar in various forms has shown a rapid growth.

**Rheumatoid Arthritis.**—Luff[13] takes the view that it is a microbial disease, appearing either as (1) an acute or sub-acute form in young persons; or (2) a chronic form in middle-aged females. In the latter we find thickening and outgrowth of cartilage or bone, but in the former these do not occur, at least in the acute stage. Sometimes the disease is primary, but it often follows other diseases and debilitating conditions. Besides a copious and liberal diet, he advocates the use of two drugs simultaneously for long periods, namely, guaiacol carbonate in doses increasing from 5 to 20 grains three times a day; and potassium iodide in 10 grain doses. With the latter tonics may be given, such as nux vomica and glycerophosphates. He is convinced that these two drugs are far superior to other remedies, and that they will in most cases arrest the disease. During the pyrexial stage of the acute form quinine is, however, preferable. Hot douches, electric light baths, and massage, both general and local, should also be employed. Llewellyn Jones reviews the very different organisms which have been found by various investigators, and suggests that various toxins may affect the nervous system and give rise to the symptoms as in the case of tetany and Raynaud's disease, in which also periarticular swellings sometimes appear. There are prodromal symptoms which also point to a primary nerve injury, such as vasomotor changes, pains, spasms, and increased reflexes and muscular atrophy, all of which may precede joint changes. Moreover these symptoms and the affections of the joints themselves sometimes appear according to the areas related to spinal segments rather than to the areas of peripheral nerves. In another paper[14] he works out the close relationship between rheumatoid arthritis and Raynaud's disease. He notes that they frequently occur in the same person. In both diseases there is often contraction of the visual field. Hæmoglobinuria and symmetrical gangrene have been found in both, and "dead fingers" sometimes precede rheumatoid arthritis. In connection with the chronic intestinal disorders which often precede rheumatoid arthritis, Helen Baldwin [15] has shown that there are evidences in the urine of perverted metabolism, such as excessive intestinal putrefaction and an abnormal amount of organic acids in the urine. Great success has been claimed for Balfour's treatment [16] by combined diet, medication, and counter irritation. Easily digested food, including white meat and fish, is ordered. Sugar, potatoes, and most vegetables are forbidden; tea is only allowed if infused in boiling milk instead of water. A little arsenic and iron is given for three weeks out of every month, and cod-liver oil whenever it can be digested. A blister is applied about once a week to each of the larger joints, and even to the smaller ones if possible, unless albuminuria occurs. This course of treatment should be continued for two or three years, and even after the disease is arrested the same diet should be continued, and the arsenic and iron taken from time to time. Orr, who describes the system, allows that other remedial agents, such as guaiacol and electricity, give great temporary benefit, but none of them, he thinks, produce the lasting and important results which are to be obtained under the Balfour method.

[12] Brit. Med Jour., July 8. [13] Practit., January. [14] Birm. Med. Rev., April. [15] Med. Rec., January 7. [16] Med. Rec. March 4.

